# Anesthetic Management of a Pediatric Patient With Wilsons Disease

**DOI:** 10.4021/jocmr2010.04.292w

**Published:** 2010-04-15

**Authors:** Mehmet Baykal, Sami Karapolat

**Affiliations:** a Department of Anesthesiology, State Hospital, Bitlis, Turkey; b Department of Thoracic Surgery, State Hospital, Bitlis, Turkey

## Abstract

Wilsons disease, characterized by cirrhosis, extrapyramidal symptoms and Kayser-Fleischer corneal rings, is a rare hereditary disease of human copper metabolism. Clinical findings in Wilsons disease are complex and neurological symptoms such as tremor, dysarthria, rigid dystonia, seizures, psychiatric disorders, acute liver failure, chronic hepatitis or cirrhosis may develop. A 4-year-old male patient was operated for traumatic depressed skull fracture and intracerebral hematoma. He was diagnosed with Wilsons disease at the age of 2.5 years and treated with zinc sulphate and D-penicillamine. General anesthesia was induced with propofol, fentanyl, atracurium, and maintained with isoflurane, and oxygen. No complications were encountered during the operation or in the postoperative period. We concluded that general anesthesia can successfully be given to Wilsons disease patients using an anesthetic agent, the metabolism of which is least affected by the liver disease, one that induces least hepatic toxicity. By close follow-up of patients clinically and biochemically, it is possible to reduce the complication rates to a minimum.

**Keywords:**

Wilson's Disease; Craniocerebral trauma; Thoracic injuries; General anesthesia; Surgery

## Introduction

Wilsons disease (WD), also known as hepatolenticular degeneration, is an autosomal recessive disorder characterized by a reduction in the synthesis of the copper transporter protein ceruloplasmin [1]. WD involves loss of the ability to export copper from the liver into bile and to incorporate copper into hepatic ceruloplasmin. Accumulation of copper in various organs and tissues occurs, especially in the liver, brain, kidneys, and cornea [2]. It can present clinically as liver disease, as a progressive neurological disorder, or as psychiatric illness. WD presents with liver disease more often in children and younger adult patients than in older adults. The type of the liver disease can be highly variable, ranging from asymptomatic with only biochemical abnormalities to acute liver failure or chronic liver disease leading to cirrhosis [3]. Due to this existing liver disease, anesthetic management presents a challenge in WD cases in terms of anesthetic technique and anesthetic drugs to be used. With this case report, we are discussing our anesthesia protocol we used during an emergency surgical procedure for trauma in a pediatric WD patient and experiences in perioperative and postoperative periods.

## Case Report

A 4-year old male patient with normal motor and mental development was brought to the emergency room where he was assessed, after falling from height. During the physical examination, hematoma of the right upper eyelid, swelling of the right half of the frontal region, and ecchymosis measuring 5 × 4 cm over infero-lateral aspect of the right hemithorax were noted. Patient was responsive, cooperated but somnolent. Cranial computed tomography revealed a depressed fracture of the right half of the frontal bone and hematoma measuring 2 × 3 cm on the lateral aspect of the right frontal lobe. Chest roentgenogram and thoracic computed tomography showed increased parenchymal density in the right paracardiac area and irregular, nodular infiltration in the right lower lobe, respectively. Family history revealed that patient's parents were related, that three of his siblings (2 brothers and 1 sister) had WD and that one brother and one sister died of liver failure. Patient has been followed-up since the age of 9 months and had liver biopsy 18 months earlier that showed minimal hepatitis. Patient did not have Kaiser-Fleischer ring and Stage I WD was diagnosed when serum alanine aminotransferase (ALT): 48 IU/L, the serum aspartate aminotransferase (AST): 47 IU/L, ceruloplasmin: 2.26 mg/dL, 24-hour urinary copper excretion: 370 μg. Patient was placed on zinc sulphate 45 mg/day and D-penicillamine 750 mg/day and this treatment was maintained without any interruptions. Preoperative tests showed Hemoglobin: 10.2 g/dL, Platelets: 272,000 /mm^3^, Na: 140 mmol/L, K: 3.8 mmol/L, BUN: 8 mg/dL, Creatine: 0.5 mg/dL, International normalized ratio (INR): 1.16, ALT: 24 IU/L, AST: 38 IU/L.

General anesthesia was induced with intravenous 2 mg/kg propofol, 2 μg/kg fentanyl, 0.5 mg/kg atracurium, and maintained with 0.8 - 1.5% isoflurane, in 50% air and 50% oxygen. ET CO_2_ was maintained between 28 - 34 mm Hg. During the operation, drainage of the hematoma and duroplasty were performed. Patient was stable hemodynamically throughout the surgery, extubated without any problems and taken to the intensive care unit. He was fully alert and responsive in postoperative 3 hours. There was no postoperative deterioration in the liver or renal functions. All of the routine laboratory tests were within the normal limits. Zinc sulphate and D-penicillamine treatment at pre-operation doses was restarted at postoperative 12 hours. No pathology was noted on chest roentgenogram taken on day 3. The patient was discharged after recovery on the day 7 of the surgery. Clinical examination and laboratory tests did not show any pathology during follow-up visit at 1 month.

## Discussion

Although WD is congenital, signs and symptoms first appear around the age of 6 years. First sign in the majority of cases, especially in children, is liver disease. When there is a positive family history and parents are related to each other, the risk among offspring of being affected is 25%. All siblings of our patient had undergone family screening in their first year of life due to history of WD. The patient who was asymptomatic was diagnosed early with WD when fine needle aspiration biopsy from the liver showed histological features of autoimmune hepatitis, serum ceruloplasmin level was low and 24-hour urinary copper excretion was significantly above its basal level. The patient was placed on medical treatment, aiming to prevent the progression of the disease and, consequently, severe sequels and organ damage in the long term and to lead a normal life.

Decrease in total hepatic blood flow which occurs normally during general anesthesia, the effects of anesthetics that are toxic to the liver, decreased blood pressure during anesthesia and decrease in tissue perfusion as a result of surgery may further disrupt hepatic function that is already impaired in WD and cause hepatic injury. On the other hand, impaired hepatic function can adversely affect the absorption, distribution, metabolism, and excretion of anesthetics, muscle relaxants, analgesics and sedatives. In fact, there is no optimal anesthetic drug in chronic liver diseases such as WD. Still, phentanyl, a narcotic agent that is least affected by these and isoflurane, an inhaled anesthetic that increases hepatic blood flow are safer choices [4].

Further, there is an increase in sensitivity to neuromuscular relaxants in WD patients. This is related to decreased muscle function due to the disease itself and D-penicillamine use over the years.

Therefore, the effect of neuromuscular blocking agents may be prolonged in patients with WD. Atracurium, which we used, is considered as first choice because it relies on neither the liver nor kidney for excretion.

A case of WD diagnosed by occurrence of acute neuropsychiatric symptoms after general anesthesia has been reported [5]. Hypnotic and sedative drugs interfere significantly with the central nervous system and may, therefore, exacerbate neurological and psychiatric problems in the postoperative period [1]. Moreover, in addition to the adverse effects of general anesthesia on the central nervous system, those related to concomitance of head trauma and neurosurgery can cause occurrence of neuropsychiatric symptoms. However, there was no known preoperative neuropsychiatric symptom in our patient and it was not observed postoperatively.

Due to their potential to trigger liver disease and cause conditions specific to WD such as hemolytic anemia, serum aminotransferases, INR and hemoglobin were closely monitored in the postoperative period and no pathology was detected.

Pulmonary contusion, which usually results from blunt trauma, consists of hemorrhage into the alveolar and interstitial spaces. Many contusions are small and contribute little to patient morbidity [6]. Treatment in such cases is usually supportive and patients heal without a sequel. Medical treatment was not given to our case since pulmonary contusion was very confined. Follow-up with arterial blood gas analysis and chest radiographs was sufficient and the patient improved clinically and radiologically in early stages.

Treatment of WD is lifelong and even a short-term, temporary break in pharmacologic therapy could result in copper accumulation. Therefore medical treatment should be reinstated as soon as possible postoperatively. Hence, we re-started medical treatment with his previous medications as soon as we ensured that oral intake would not cause a problem.

In conclusion, general anesthesia can be successfully achieved in an asymptomatic, pediatric case of WD using agents that are least toxic to the liver. Preventive measures and meticulous observation and follow-up in the perioperative and postoperative periods would minimize the complication rates and secure a successful outcome in WD cases.
